# DNA methylation mediates MBSR induced cardioprotection in patients after PCI

**DOI:** 10.1038/s41598-026-51616-6

**Published:** 2026-05-09

**Authors:** Mengyuan Xiong, Xingkui Dou, Jifa Tao, Fei Hu, Pan Jing, Zhao Zhao, Hongyan Cai, Zhao Hu, Min Zhang

**Affiliations:** 1 Cardiology Department, The First Affiliated Hospital of Kunming Medical University, Kunming, 650032 China; 2 Cardiology Department, The Second Affiliated Hospital of Kunming Medical University, Kunming, 650101 China; 3 Geriatric Cardiology Department, The First Affiliated Hospital of Kunming Medical University, Kunming, 650032 China

**Keywords:** Mindfulness-based stress reduction, DNA methylation, Acute myocardial infarction, Inflammation, Cardiac function, Biomarkers, Cardiology, Diseases, Medical research

## Abstract

Mindfulness-Based Stress Reduction (MBSR) improves well-being in acute myocardial infarction (AMI) patients, but its molecular cardioprotective mechanisms are unclear. In this prospective pilot study, 51 post-PCI AMI patients (17 MBSR plus standard therapy, 34 controls) were assessed for inflammatory markers (CRP, IL-6, PCT), echocardiography, and functional capacity at baseline and 6 months. Genome-wide DNA methylation profiling in a subgroup (*n* = 5/group) assessed changes from baseline to 6 months within MBSR and compared groups at the 6-month follow-up. At follow-up, the MBSR group showed greater reductions in CRP, IL-6, and PCT (adjusted *P* = 0.001, < 0.001, 0.003), alongside improved left ventricular ejection fraction, global longitudinal strain, wall motion score index, and 6-minute walk distance; all remained significant after false discovery rate correction (all q < 0.05). MBSR induced widespread DNA methylation changes (7,665 intra-group and 7,791 inter-group differentially methylated sites), which were nominally enriched in pathways related to immune response, inflammation, oxidative stress, and neuroendocrine (HPA axis) regulation. Exploratory uncorrected analysis linked BRD4 hypermethylation with lower CRP (ρ=-0.601) and improved GLS (ρ = 0.802), and PTTG1IP hypomethylation with improved LVEF (ρ = 0.717). These findings suggest MBSR modulates the DNA methylome within a novel “epigenetic-inflammatory-cardiac functional axis”, providing a mechanistic framework for its cardioprotective effects.

**Supplementary Information:**

The online version contains supplementary material available at 10.1038/s41598-026-51616-6.

## Introduction

Acute myocardial infarction (AMI) represents a critical global health burden, influenced by psychosocial stress pathways^1^. While percutaneous coronary intervention (PCI) restores coronary flow, procedural trauma and disease uncertainty trigger psychological distress^2^. Post-AMI anxiety and depression are well-recognized predictors of adverse cardiovascular outcomes, including recurrent events and increased mortality^3^, underscoring the urgent need for effective psychological interventions. Interventions mitigating post-AMI stress improve mental well-being and long-term clinical prognosis^4^, providing a compelling rationale for integrating mind-body therapies into standard cardiac care.

Mindfulness-Based Stress Reduction (MBSR), a standardized 8-week program by Kabat-Zinn^5^, cultivates present-moment awareness through meditation and yoga. Emerging evidence supports its utility in AMI populations, benefiting reduction of negative emotions^6^ and lower post-PCI cardiovascular event rates^7^. However, the molecular mechanisms underpinning these cardioprotective effects remain incompletely understood. Most studies rely on subjective psychological metrics, lacking objective biomarkers to clarify pathophysiology.

Recent research suggests contemplative practices induce epigenetic modifications, particularly in stress-responsive genes^8^. DNA methylation serves as a pivotal interface linking psychological stress to inflammatory-immune dysregulation, oxidative stress, and maladaptive cardiac remodeling^9,10^. Specifically, AMI provokes detrimental methylation changes in inflammation- and HPA axis-related genes (e.g., NOD-like receptor signaling)^11,12^, critical drivers of post-infarct myocardial injury and heart failure progression^13,14^. We thus hypothesize that mindfulness-based interventions like MBSR confer cardioprotection by counteracting these maladaptive epigenetic programs.

To address this gap, this prospective matched-control pilot study explored the mechanisms by integrating genome-wide DNA methylation profiling with serial assessments of inflammatory biomarkers, echocardiographic parameters, and objective functional measures (6-minute walk test) in post-PCI AMI patients. We primarily aimed to explore whether MBSR induces dynamic changes along a potential “epigenetic-inflammatory-cardiac functional axis”. Specifically, we sought to test the hypothesis that MBSR-induced DNA methylation alterations in key pathways are associated with reduced inflammation and improved cardiac/physical function, thereby providing a preliminary molecular framework for its therapeutic benefits.

## Materials and methods

**Fig. 1:**
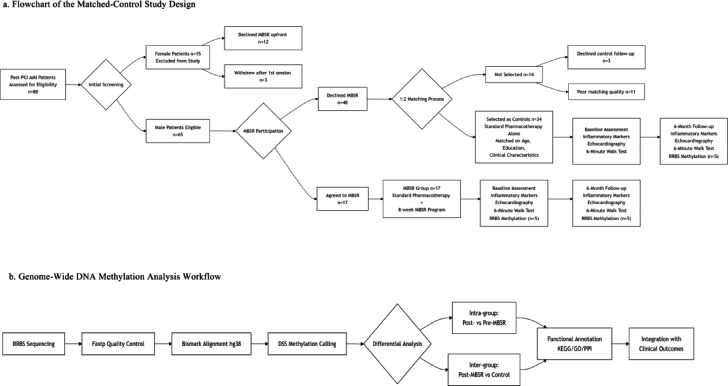
Study design and molecular analysis workflow. **(a)** Flowchart of the matched-control study design. **(b)** Genome-wide DNA methylation analysis workflow.

### Study design and participant recruitment

This prospective, matched-control pilot study was registered at the Chinese Clinical Trial Registry (ChiCTR2500113074). The study was conducted over 24 months (January 2022 to December 2023) at cardiology departments of the First and Second Affiliated Hospitals of Kunming Medical University, China. Its protocol was approved by the Ethics Committee of the First Affiliated Hospital of Kunming Medical University (Approval No. (2021) L-126), and all participants provided written informed consent. All study procedures were conducted in accordance with the Declaration of Helsinki and relevant guidelines and regulations. To maintain ethical equipoise, control group patients were offered the opportunity to participate in a similar MBSR program upon completion of the 6‑month follow‑up assessments.

The overall design and participant flow are illustrated in Fig. 1a. A total of 80 consecutive post-AMI patients were assessed for eligibility. Following screening, all 15 eligible female patients declined to participate in the MBSR program (12 declined upfront and 3 withdrew after the first session), resulting in 65 male patients. From this pool, 17 patients constituted the MBSR intervention group after providing consent for the 8-week MBSR program plus standard pharmacotherapy. To form a comparable control group, we aimed to match controls to the MBSR participants in a 1:2 ratio based on age, educational level, and key clinical characteristics. From the remaining 48 male patients, we identified 34 who constituted the optimal matches to the intervention group. The other 14 patients were not included in the final cohort: 3 declined to participate in the follow-up assessments as controls, and 11 were not selected due to poorer matching quality with the intervention group on the predefined criteria. This process resulted in a final matched cohort of 51 patients (17 MBSR; 34 control). All participants underwent evaluations of inflammatory markers, echocardiographic parameters, and the 6-minute walk test (6MWT) at baseline and 6-month follow-up. From this cohort, a subgroup (*n* = 5 per group) was randomly selected for genome-wide DNA methylation analysis. For the MBSR group, samples were taken at baseline and after the 6-month program. For the control group, the sample was taken at the 6-month follow-up visit, matching the timing of the post-MBSR assessment (as shown in Fig. 1a). All clinical procedures were conducted within the Cardiology Departments of the participating hospitals, while genomic sequencing was performed at the Kunming Kingmed Medical Laboratory.

As a pilot mechanistic study focused on epigenetic mechanisms, a formal a priori power calculation was not performed. The total sample size (*n* = 51) was determined based on practical constraints, including the availability of eligible post-PCI AMI patients within the study period and the high cost of genome-wide methylation sequencing. The 1:2 allocation (MBSR: Control) was adopted to efficiently utilize the available MBSR participants while forming a robust comparison group through matching. The subset size for methylation analysis (*n* = 5 per group) is consistent with sample sizes reported in comparable pilot epigenetic studies. Post-hoc analysis confirmed that the large effect sizes observed in inflammatory markers, echocardiographic parameters, and 6MWT data provided sufficient statistical power to detect clinically meaningful differences between groups.

Inclusion criteria required patients aged 18–70 years, diagnosed with ST-segment elevation myocardial infarction (STEMI) or non-ST-segment elevation myocardial infarction (NSTEMI) per the Fourth Universal Definition of Myocardial Infarction^15^, who underwent emergency PCI within 24 h of symptom onset and were clinically stable one month post-PCI. Participants also needed a minimum of high school education, intact cognitive function (Mini-Mental State Examination score ≥ 24), and willingness to provide informed consent. Exclusion criteria encompassed severe comorbidities (e.g., NYHA class IV heart failure, end-stage renal disease, active malignancy), acute infection within the past 3 months, autoimmune diseases, history of immunosuppressive therapy, prior experience with structured mind-body interventions, major psychiatric disorders, severe depression, anticipated non-compliance, and any condition deemed unsuitable by the investigators.

### MBSR intervention

The MBSR group participated in an 8-week standardized MBSR program led by a certified instructor (Supplementary Table S1), in addition to receiving standard medical therapy. The program consisted of weekly 2–2.5 h group sessions, incorporating mindfulness practices such as body scanning, mindful breathing, gentle yoga, and guided discussions on stress perception and management, aligning with the classic curriculum developed by Kabat-Zinn^16^. Participants were instructed to perform daily 30-minute audio-guided mindfulness exercises at home.

Upon completion of the program, semi-structured interviews were conducted privately by a mindfulness instructor to qualitatively assess the intervention’s impact on quality of life, mindfulness skills, and self-regulatory capacity, using key questions detailed in Supplementary Table S2, adapted from Hwang et al^17^.. With participants’ consent, interviews were recorded, transcribed verbatim, and analyzed by two independent researchers using thematic analysis.

### Clinical data and biological sample collection

#### Demographic and clinical data

Demographic and clinical data, including age, body mass index (BMI), cardiac event type (STEMI/NSTEMI), Killip class, comorbidities, medication use, and procedural details, were retrieved from electronic medical records.

#### Blood sampling and inflammatory marker assays

Fasting peripheral blood samples were collected from all participants. Plasma levels of C-reactive protein (CRP), interleukin-6 (IL-6), and procalcitonin (PCT) were quantified using standard commercial immunoassays. Detailed procedures for sample processing and assay specifications are provided in the Supplementary Methods.

#### Echocardiographic assessment

Transthoracic echocardiography was performed using a Philips EPIQ7 system by certified sonographers blinded to group assignment. Standard parasternal and apical views were acquired over three cardiac cycles. Key parameters included:

- Left ventricular ejection fraction (LVEF) calculated via Simpson’s biplane method.
- Global longitudinal strain (GLS) derived from speckle-tracking echocardiography using a 17-segment model.
- Interventricular septal thickness (IVST) and left ventricular end-diastolic diameter (LVEDD) measured in the parasternal long-axis view.
- Mitral E/e’ ratio obtained from tissue Doppler imaging.
- Wall motion score index (WMSI) assessed by grading 17 left ventricular segments.

All analyses were performed offline by a single experienced observer. Intraclass correlation coefficients (ICC > 0.90) confirmed low inter-observer variability.

#### 6MWT

Functional exercise capacity was objectively assessed using the standardized 6MWT, performed in accordance with the American Thoracic Society guidelines^18^ at both baseline and the 6-month follow-up. Participants were instructed to walk as far as possible along a marked, flat, 30-meter hospital corridor over 6 min. Standardized verbal encouragement was given at regular intervals. The total distance walked, measured in meters, was recorded. The test was administered by research staff who were blinded to the participants’ group allocation. All tests were conducted under the supervision of a study cardiologist to ensure patient safety.

### DNA methylation profiling and bioinformatics analysis

Genome-wide DNA methylation profiling was performed on a random subgroup (*n* = 5 per group) using Reduced Representation Bisulfite Sequencing (RRBS)^19^. The overall bioinformatic workflow is summarized in Fig. 1b.

Genomic DNA was extracted from buffy coat cells. The average sequencing depth was ≥ 10× for all samples, meeting the recommended coverage for reliable methylation calling. After quality control and filtering, approximately 3.2 million high-quality CpG sites were retained for downstream analysis. To minimize potential confounding by genetic variation, CpG sites overlapping with known SNPs (dbSNP build 155) were excluded. RRBS libraries were prepared, subjected to bisulfite conversion, and sequenced on an Illumina NovaSeq 6000 platform (PE150). Detailed laboratory protocols are provided in the Supplementary Methods.

Bioinformatic analysis followed a standard pipeline. Briefly, raw sequencing reads underwent quality control and adapter trimming before alignment to the human reference genome (hg38) using Bismark^20^. Differential methylation analysis was performed with the DSS package^21^ to identify differentially methylated sites (DMS) and regions (DMR), using an FDR threshold of < 0.05 and an absolute methylation difference ≥ 3%.

RRBS is well‑suited for exploratory pilot studies with limited sample sizes. Recent reports have demonstrated its feasibility in small cohorts: Benincasa et al. successfully employed RRBS in 12 heart failure patients (8 ischemic vs. 4 non‑ischemic) to distinguish etiological subtypes with high accuracy (AUC > 0.8)^22^, and Gittens et al. identified significant differential methylation associated with prenatal tobacco smoke exposure in 16 preterm infants (7 exposed, 9 controls) using RRBS^23^. These findings support the sensitivity and utility of RRBS for hypothesis‑generating methylation discovery in modestly sized samples.

### Statistical and bioinformatics analysis

#### Analysis of clinical outcomes

Continuous variables are presented as mean ± standard deviation (SD) or median (interquartile range, IQR) as appropriate; categorical variables as counts (%). Baseline characteristics were compared using Student’s t‑test, Mann‑Whitney U test, χ² test, or Fisher’s exact test, as appropriate.

For longitudinal analyses, within‑group changes (baseline to 6 months) were assessed with paired t‑tests (normally distributed data) or Wilcoxon signed‑rank tests (non‑normally distributed data). The primary analysis focused on between‑group differences at 6‑month follow‑up. To control for potential confounding, adjusted models were used: a Generalized Linear Model (GLM) with Gamma distribution and log link for inflammatory markers (CRP, IL‑6, PCT), and Analysis of Covariance (ANCOVA) for normally distributed outcomes (echocardiographic parameters and 6MWD). All models adjusted for the baseline value of the respective outcome and for baseline covariates that differed between groups (*P* < 0.05; see Results, Table 1). Statistical significance was set at a two‑tailed *P* < 0.05.

To account for multiple comparisons across the multiple outcome measures, we applied the Benjamini‑Hochberg false discovery rate (FDR) procedure separately to the adjusted between‑group P‑values of the three inflammatory markers and the five prespecified cardiac/functional outcomes (LVEF, GLS, WMSI, IVST, and 6MWD; Table 2). An FDR q‑value < 0.05 was considered statistically significant for these exploratory analyses.

#### Functional enrichment analysis

Differential methylation data were analyzed to identify enriched biological functions and pathways. Standard bioinformatics pipelines were employed for functional annotation (Gene Ontology, GO), pathway analysis (Kyoto Encyclopedia of Genes and Genomes, KEGG), and protein-protein interaction (PPI) network construction. The GSE208194 dataset^24^, derived from plasma of patients with coronary artery disease, was chosen for cross‑validation because it represents a relevant, publicly available transcriptomic resource from a clinically similar population, allowing us to generate a prioritized list of hub genes for exploratory analysis. Details of software packages and analytical parameters are provided in the Supplementary Methods. An exploratory correlation analysis between hub gene methylation and clinical phenotypes was performed using Spearman’s rank test, with P-values adjusted for multiple comparisons via the Benjamini-Hochberg FDR procedure. An FDR < 0.10 was considered significant for these hypothesis-generating analyses.

## Results

### Characteristics of subjects at baseline

A total of 51 patients were included with the MBSR group (*n* = 17) and control group (*n* = 34) having average ages of 53.6 ± 6.1 years and 54.9 ± 9.2 years, respectively. Baseline characteristics were comparable between groups, except for a significant difference in GP IIb/IIIa inhibitor use (*P* = 0.005) (Supplementary Table S3).

Due to the significant between-group difference in the use of GP IIb/IIIa inhibitor, this variable was included as a covariate in all subsequent adjusted analyses (GLM and ANCOVA models) of 6-month outcomes, in addition to the baseline values of the respective outcome measures.

### Effects of MBSR on inflammatory markers

Longitudinal data for inflammatory markers were available for 15 participants in the MBSR group and 28 in the control group at 6‑month follow‑up, as two and six participants declined the follow‑up blood draw. All analyses of inflammatory markers are based on these available data. At baseline, the MBSR and control groups exhibited comparable inflammatory profiles (all *P* > 0.05). At 6‑month follow‑up, CRP, IL‑6, and PCT levels were significantly lower in the MBSR group compared to the control group in unadjusted analyses (*P* = 0.003, *P* = 0.043, and *P* = 0.038, respectively; Table 1). Within‑group analyses revealed significant reductions from baseline to 6 months in the MBSR group for all three inflammatory markers (CRP: *P* = 0.028; IL‑6: *P* < 0.001; PCT: *P* = 0.033), whereas no significant changes were observed in the control group (all *P* > 0.05). After adjusting for baseline levels and GP IIb/IIIa inhibitor use using GLM, the between‑group differences at 6 months remained highly significant (CRP: adjusted *P* = 0.001; IL‑6: adjusted *P* < 0.001; PCT: adjusted *P* = 0.003). Applying Benjamini‑Hochberg FDR correction for multiple comparisons across the three inflammatory markers, all three adjusted P‑values yielded an FDR q‑value of 0.003 (Table 1), confirming that the reductions in CRP, IL‑6, and PCT remained statistically significant after correction. These results support a specific effect of MBSR in reducing systemic inflammation.

**Table 1:** Changes in inflammatory markers over time (with FDR adjustment).

Inflammatory marker [M (Q1, Q3)]	MBSR group (*n* = 15)	Control group (*n* = 28)	Unadj. intergroup *P*-value ¹	Adj. intergroup *P*-value ²	FDR q-value ³
**CRP (mg/L)**					
Baseline	0.70 (0.20, 5.74)	2.10 (0.90, 3.20)	0.335	-	-
6-Month Follow-up	0.30 (0.15, 0.80)	1.30 (0.60, 3.29)	0.003	0.001	0.003
Intra-group P-value ⁴	0.028	0.936			
**IL-6 (pg/mL)**					
Baseline	5.33 (2.59, 11.89)	4.25 (2.30, 6.86)	0.224	-	-
6-Month Follow-up	2.20 (1.20, 3.50)	3.30 (2.30, 7.22)	0.043	< 0.001	0.003
Intra-group P value ⁴	< 0.001	0.677			
**PCT (ng/mL)**					
Baseline	0.05 (0.03, 0.07)	0.05 (0.05, 0.05)	0.323	-	-
6-Month Follow-up	0.04 (0.02, 0.06)	0.05 (0.05, 0.05)	0.038	0.003	0.003
Intra-group P value ⁴	0.033	1			

Note:

^1^ Unadjusted intergroup P-value: Mann-Whitney U test.

² Adjusted intergroup P-value: Generalized linear model (GLM, Gamma distribution, log link), adjusted for baseline values and use of GP IIb/IIIa inhibitors.

³ FDR q-value: Multiple comparison correction was performed for the 3 adjusted intergroup P-values in the table using the Benjamini–Hochberg method with Q = 0.05. All q-values were < 0.05.

⁴ Intra-group P-value: Wilcoxon signed-rank test (baseline vs. 6 months).

CRP: C-reactive protein; IL-6: interleukin-6; PCT: procalcitonin.

### Effects of MBSR on cardiac and functional outcomes

All 51 enrolled participants completed both baseline and 6‑month echocardiographic assessments (Table 2). At baseline, the MBSR group had significantly smaller LVEDD and IVST than the control group (*P* = 0.024 and *P* = 0.007, respectively). At 6‑month follow‑up, significant within‑group improvements were observed in the MBSR group for LVEF (52.9% to 57.5%, *P* < 0.05), GLS (−14.3% to −15.9%, *P* < 0.05), and WMSI (1.1 to 1.0, *P* < 0.05), indicating enhanced cardiac function and attenuated remodeling. In unadjusted between‑group comparisons at 6 months, the MBSR group demonstrated significantly better LVEF, GLS, and WMSI, and lower IVST compared to the control group (all *P* < 0.05). After adjusting for baseline values and GP IIb/IIIa inhibitor use, the superior outcomes in the MBSR group for LVEF (adjusted *P* = 0.002), GLS (adjusted *P* = 0.027), WMSI (adjusted *P* = 0.029), and IVST (adjusted *P* = 0.039) remained statistically significant. To control for multiple comparisons, we applied the Benjamini‑Hochberg FDR correction to the adjusted P‑values of LVEF, GLS, WMSI, 6MWD, and IVST (five prespecified outcomes). All five remained significant with FDR q‑values as follows: LVEF (q = 0.010), GLS (q = 0.045), WMSI (q = 0.045), 6MWD (q = 0.010), and IVST (q = 0.045) (Table 2). These results robustly demonstrate the cardioprotective effect of MBSR independent of baseline characteristics.

Functional exercise capacity, assessed by the 6‑minute walk distance (6MWD), is also summarized in Table 2. At baseline, the two groups showed comparable capacity, with no significant difference in 6MWD (MBSR: 436.7 ± 38.5 m vs. Control: 449.0 ± 35.9 m; *P* = 0.26). At 6‑month follow‑up, both groups demonstrated significant within‑group improvements from baseline (both *P* < 0.05). However, the improvement was greater in the MBSR group (523.1 ± 30.5 m) compared to the control group (496.9 ± 23.0 m). This between‑group difference was statistically significant in both unadjusted analysis (*P* = 0.001) and after adjustment for baseline 6MWD and GP IIb/IIIa inhibitor use (adjusted *P* = 0.003), and remained significant after FDR correction (q = 0.010; Table 2).

**Table 2:** Changes in cardiac function and functional outcomes from baseline to 6 month follow up (with FDR adjustment).

Parameter (mean ± SD)	Time point	MBSR group (*n* = 17)	Control group (*n* = 34)	Unadj. intergroup *P*-value ¹	Adj. intergroup *P*-value ²	FDR q-value ³
LVEDD (mm)	Baseline	44.7 ± 3.5	48.5 ± 6.4	0.02 ^†^	-	-
	6-Month	45.6 ± 5.9	49.5 ± 7.0	0.05	0.94	-
IVST (mm)	Baseline	11.2 ± 2.0	12.8 ± 1.9	0.007 ^†^	-	-
	6-Month	11.1 ± 1.6	12.8 ± 2.0	0.002 ^†^	0.04	0.045
E/e′	Baseline	9.4 ± 2.0	10.6 ± 3.1	0.13	-	-
	6-Month	10.1 ± 3.3	10.8 ± 2.9	0.41	-	-
FS (%)	Baseline	33.6 ± 6.9	33.9 ± 8.5	0.89	-	-
	6-Month	32.8 ± 5.6	33.6 ± 7.5	0.7	-	-
LVEF (%)	Baseline	52.9 ± 7.2	52.1 ± 12.0	0.81	-	-
	6-Month	57.5 ± 5.7 ^*^	51.5 ± 10.3	0.03 ^†^	0.002	0.01
GLS (%)	Baseline	−14.3 ± 3.3	−15.0 ± 3.2	0.47	-	-
	6-Month	−15.9 ± 3.0 ^*^	−13.9 ± 3.3	0.03 ^†^	0.027	0.045
WMSI	Baseline	1.1 ± 0.1	1.2 ± 0.3	0.29	-	-
	6-Month	1.0 ± 0.1 ^*^	1.1 ± 0.2	0.03 ^†^	0.029	0.045
6MWD (m)	Baseline	436.7 ± 38.5	449.0 ± 35.9	0.26	-	-
	6-Month	523.1 ± 30.5 ^*^	496.9 ± 23.0 ^*^	0.001 ^†^	0.003	0.01

Note:

^*^ Significant intra-group difference (6 months vs. baseline) by paired t-test, *P* < 0.05.

^†^ Significant unadjusted inter-group difference by independent samples t-test, *P* < 0.05.

¹ Unadjusted inter-group P-value: independent samples t-test.

² Adjusted inter-group P-value: ANCOVA adjusted for baseline values and use of GP IIb/IIIa inhibitors. ANCOVA was only performed if unadjusted *P* < 0.05.

³ FDR q-value: Multiple comparison correction was applied to all 5 adjusted inter-group P-values in the table (LVEF, GLS, WMSI, 6MWD, IVST) using the Benjamini–Hochberg method with Q = 0.05. All q-values were < 0.05.

“-” indicates no ANCOVA performed (baseline comparison) or no adjustment performed (due to unadjusted *P* > 0.05).

LVEDD: Left Ventricular End-Diastolic Diameter; IVST: Interventricular Septal Thickness; E/e’: E-wave to e’-wave ratio; FS: Fractional Shortening; LVEF: Left Ventricular Ejection Fraction; GLS: Global Longitudinal Strain; WMSI: Wall Motion Score Index; 6MWD: 6-minute walk distance.

LVEF was measured using Simpson’s biplane method (apical four-chamber and two-chamber views) instead of the Teichholz formula, which assumes a geometric model of left ventricular geometry.

### Genome-wide DNA methylation alterations

#### Reliability of methylation profiles

**Fig. 2:**
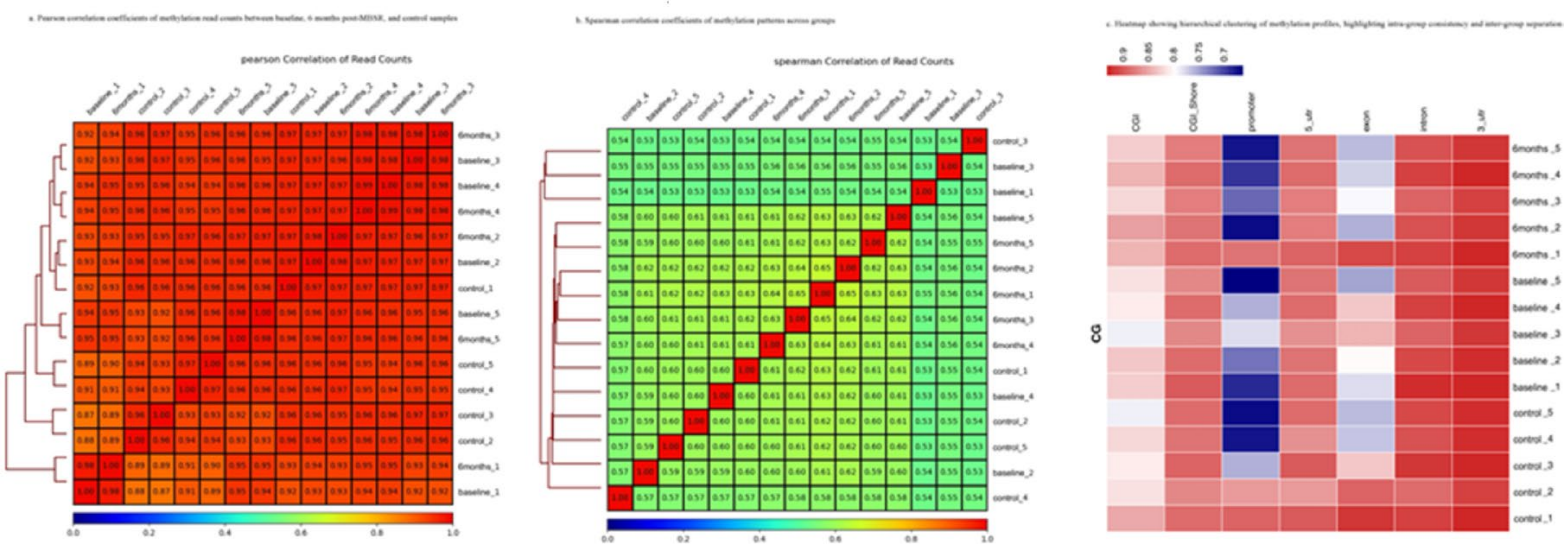
Reliability of DNA Methylation Profiling across Groups. **(a)** Pearson correlation coefficients of methylation read counts between baseline, 6 months post-MBSR, and control samples; **(b)** Spearman correlation coefficients of methylation patterns across groups; **(c)** Heatmap showing hierarchical clustering of methylation profiles, highlighting intra-group consistency and inter-group separation.

Technical analysis confirmed high reproducibility of the DNA methylation data. Methylation patterns showed strong intra‑group consistency and clear inter‑group separation across time points, as evidenced by Pearson and Spearman correlation analyses (Fig. 2a and b) and hierarchical clustering of methylation profiles across all samples (Fig. 2c). These results collectively indicate robust sequencing quality and biological relevance for downstream analysis. These correlation metrics are presented as quality control indicators for sequencing reproducibility rather than as direct evidence of biological similarity.

Furthermore, the distribution of DNA methylation Beta values for high-quality CpG sites (depth ≥ 10×) exhibited a typical bimodal distribution, consistent with the well-characterized biological features of the human peripheral blood cell methylome (Supplementary Fig. S1). Strict quality control filtering, including the removal of low-quality reads, adapter sequences, and CpG sites overlapping known SNPs, further validated the reliability of the dataset for subsequent differential methylation and enrichment analyses.

#### Differentially methylated sites (DMS) and regions (DMR)

MBSR induced widespread nominal alterations in the DNA methylome. Comparing post- to pre-MBSR samples (intra-group), we identified 7,665 differentially methylated sites (DMS) and 206 differentially methylated regions (DMR) under exploratory criteria (FDR < 0.05, |Δmethylation| ≥ 3%). In the inter-group comparison (post-MBSR vs. control), 7,791 DMS and 217 DMR were identified. Both DMS and DMR were predominantly located in gene bodies and intergenic regions, with a smaller fraction in promoter areas (Supplementary Fig. S2). This distribution is functionally relevant: gene body methylation is often associated with active transcription and alternative splicing, intergenic methylation can mark enhancer elements, and promoter methylation typically represses transcriptional initiation. Thus, MBSR-induced changes across these compartments may coordinately modulate stress-responsive and inflammatory pathways.

After applying strict genome-wide multiple testing correction (FDR < 0.05), no DMS or DMR remained significant (Supplementary Table S4). Given the pilot and exploratory nature of this study, subsequent pathway enrichment analyses were conducted using the nominal DMS/DMR sets to identify potential biological themes, acknowledging that these findings are hypothesis-generating.

### Functional enrichment analysis of differentially methylated genes

Functional analysis of genes associated with differential methylation revealed significant enrichment in pathways governing stress response, inflammation, immunity, and cardiac remodeling.

#### Pathway enrichment analysis

**Fig. 3:**
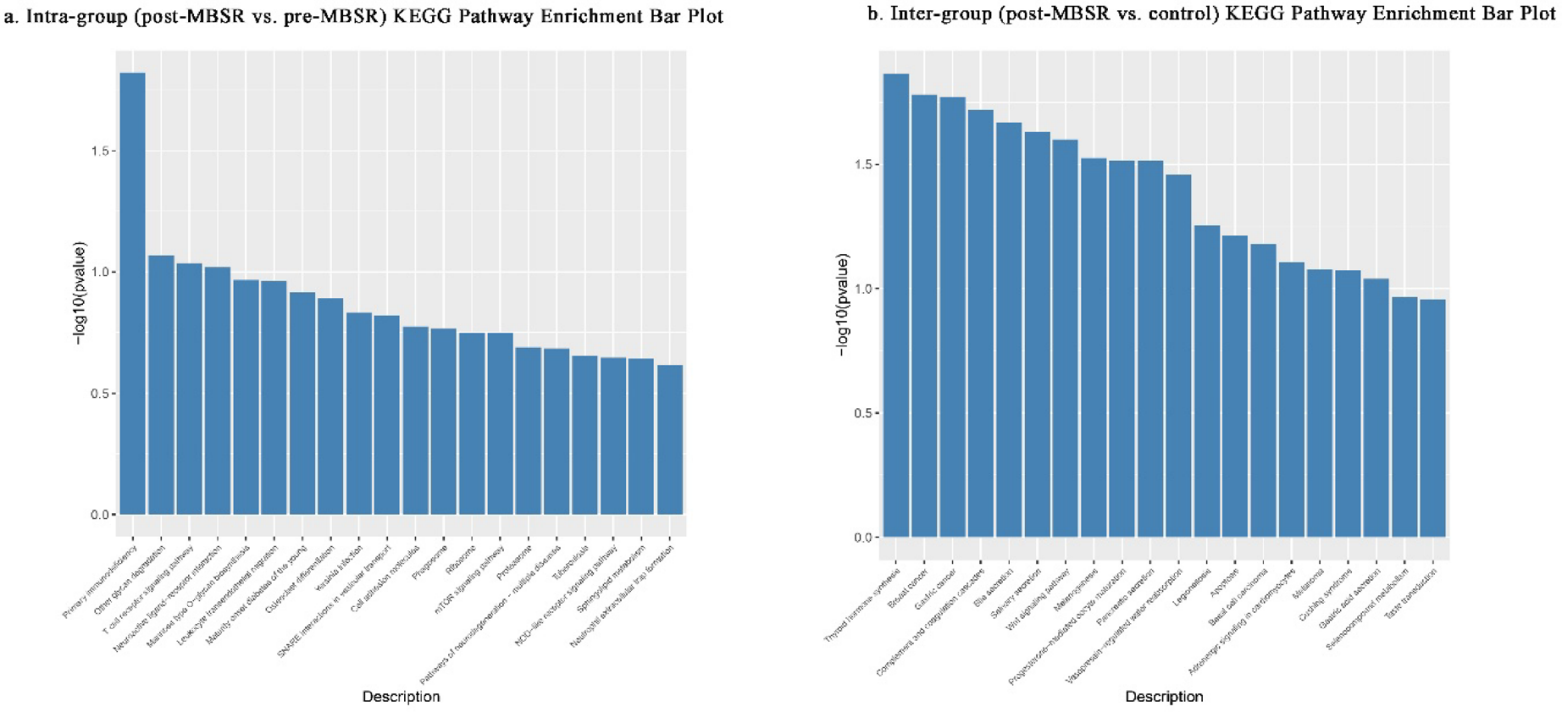
KEGG Pathway Enrichment Analysis. (a) Intra-group (post-MBSR vs. pre-MBSR) KEGG Pathway Enrichment Bar Plot; (b) Inter-group (post-MBSR vs. control) KEGG Pathway Enrichment Bar Plot. Pathways shown are those with nominal enrichment (raw *P* < 0.1) before multiple comparison correction. After Benjamini‑Hochberg FDR correction, no pathway remained statistically significant (all padj > 0.05). KEGG pathway maps were generated with permission from Kanehisa Laboratories^25^.

To explore potential biological themes, we performed KEGG pathway enrichment analysis using the nominal DMS/DMR sets (FDR < 0.05, |Δmethylation| ≥ 3%, uncorrected for multiple comparisons across pathways). Within the MBSR group (intra-group comparison, post-MBSR vs. baseline), several pathways relevant to stress response and cardiovascular function showed nominal enrichment (raw *P* < 0.1), including neuroactive ligand‑receptor interaction (raw *P* = 0.0958), T‑cell receptor signaling pathway (raw *P* = 0.092), and primary immunodeficiency (raw *P* = 0.015). In the inter‑group comparison (post-MBSR vs. control), pathways such as thyroid hormone synthesis (raw *P* = 0.0136), complement and coagulation cascades (raw *P* = 0.019), Wnt signaling pathway (raw *P* = 0.0251), breast cancer (raw *P* = 0.0165), and gastric cancer (raw *P* = 0.0169) exhibited raw P‑value trends below 0.1. These nominally enriched pathways are visualized in Fig. 3a (intra-group) and Fig. 3b (inter-group). After Benjamini‑Hochberg false discovery rate (FDR) correction for multiple comparisons across all tested pathways, no KEGG pathway reached statistical significance (all padj > 0.05). The complete, unfiltered KEGG enrichment results are provided in Supplementary Table S5 and Supplementary Data 1.

Representative nominally enriched Gene Ontology (GO) terms are visualized in Supplementary Fig. S3. GO analysis of the nominal DMS/DMR sets revealed raw P‑value trends relevant to inflammation, oxidative stress, and neuroendocrine function. In the intra‑group comparison (post-MBSR vs. baseline), key nominally enriched biological processes (BP) included tRNA methylation (raw *P* = 0.0009), regulation of respiratory burst (raw *P* = 0.0026), and neurotransmitter secretion (raw *P* = 0.0046). For the inter‑group comparison (post-MBSR vs. control), nominal enrichment was observed for response to oxidative stress (raw *P* = 0.0722) and positive regulation of stress‑activated MAPK cascade (raw *P* = 0.0416). A complete list of these nominally enriched BP terms (raw *P* < 0.1) is provided in Supplementary Table S6. No GO term (BP, cellular component [CC], or molecular function [MF]) reached statistical significance after Benjamini‑Hochberg FDR correction (all padj > 0.05), and no CC terms met the nominal threshold. The full unfiltered GO enrichment results (covering BP/CC/MF) are available in Supplementary Data 1. These nominal observations align with the exploratory, hypothesis‑generating nature of the study, linking MBSR to potential epigenetic effects on stress and neuroendocrine pathways.

#### Integrated methylome-transcriptome and network analysis

To delineate the functional architecture of the methylation changes, we performed sequential network and integrated methylome-transcriptome analyses.

**Fig. 4:**
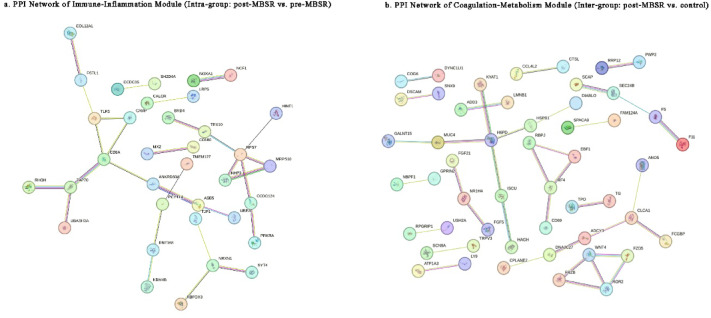
Protein-Protein Interaction (PPI) Networks of Differentially Methylated Genes. (a) PPI Network of Immune-Inflammation Module (Intra-group: post-MBSR vs. pre-MBSR). (b) PPI Network of Coagulation-Metabolism Module (Inter-group: post-MBSR vs. control).

**Fig. 5:**
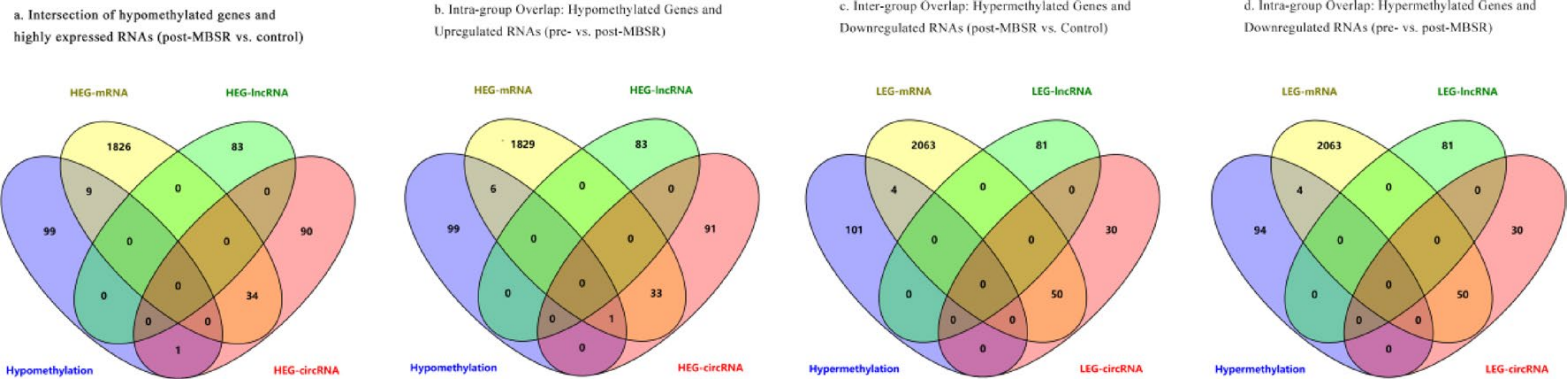
Venn Diagram Analysis of Methylome-Transcriptome Overlap. **(a)** Inter-group Overlap: Hypomethylated Genes and Upregulated RNAs (post-MBSR vs. Control. **(b)** Intra-group Overlap: Hypomethylated Genes and Upregulated RNAs (pre- vs. post-MBSR). **(c)** Inter-group Overlap: Hypermethylated Genes and Downregulated RNAs (post-MBSR vs. Control). **(d)** Intra-group Overlap: Hypermethylated Genes and Downregulated RNAs (pre- vs. post-MBSR).

Protein-protein interaction (PPI) network analysis revealed that the differentially methylated genes coalesced into two major functional modules (Fig. 4): an Immune-Inflammation module (core nodes: TLR5, CD8A, CAMP) and a Coagulation-Metabolism module (core nodes: F5, F11, ISCU, HPD) (Supplementary Table S7: Section A). Subsequently, integration of our methylome with a coronary artery disease transcriptome (GSE208194)^24^ prioritized 25 candidate hub genes (e.g., WNT4, FSTL1, NCF1, BRD4, PTTG1IP) and delineated their interaction sub networks (Supplementary Fig. S4), which distinguished inter-group and intra-group regulatory focuses (Supplementary Table S7: Section B). This integrated methylome-transcriptome analysis revealed a significant concordance: hypomethylated loci were strongly associated with transcriptomic upregulation in pathways related to immune activation and antioxidant defense (Fig. 5).

### Association of key genes with clinical phenotypes

To explore potential mechanistic links, we performed a hypothesis-generating correlation analysis between DNA methylation levels of hub genes (identified via methylome-transcriptome integration) and clinical phenotypes. After multiple comparison correction (FDR control), no statistically significant associations were identified between any gene-phenotype pairs at the pre-specified threshold (FDR < 0.10). However, in the uncorrected exploratory analysis, methylation of BRD4 was associated with lower C-reactive protein (CRP) (ρ = −0.601, raw *P* = 0.032) and better Global Longitudinal Strain (GLS) (ρ = 0.802, raw *P* = 0.030). Concurrently, methylation of PTTG1IP was associated with higher Left Ventricular Ejection Fraction (LVEF) (ρ = 0.717, raw *P* = 0.045). These uncorrected associations provide preliminary, hypothesis-generating clues for future investigation but require validation in larger, independent cohorts. The complete correlation results for all 25 hub genes are provided in Supplementary Data 2.

## Discussion

This pilot mechanistic study provides novel exploratory evidence that an 8-week MBSR program induces extensive DNA methylation reprogramming in post-PCI AMI patients, changes robustly associated with attenuated systemic inflammation and improved cardiac and functional outcomes. After rigorous adjustment for baseline confounders, the MBSR group demonstrated superior 6-month outcomes, including marked reductions in CRP, IL-6, and PCT and concurrent improvements in LVEF, GLS, WMSI, and 6MWD. The large effect sizes, observed despite the modest sample size inherent to a pilot study, underscore the potential biological potency of MBSR and provide a compelling rationale for large-scale randomized controlled trials. We acknowledge that using peripheral blood DNA methylation as a proxy for myocardial tissue is an inferential bridge. However, circulating leukocytes are central mediators of systemic inflammation and are directly responsive to neuroendocrine signals. MBSR‑induced epigenetic changes in these cells are not merely a surrogate; they may represent a functional component of the systemic anti‑inflammatory and anti‑oxidative shift. This shift, as evidenced by reduced CRP, IL‑6, and PCT, is well‑established to benefit myocardial recovery post‑AMI^9,10,26^. Therefore, the observed methylomic changes in blood likely reflect, and contribute to, the systemic environment that enables cardiac tissue repair.

To comprehensively explore the biological themes suggested by the methylation changes, we performed unbiased KEGG and GO enrichment analyses using nominally significant (raw *P* < 0.1) DMS/DMR sets. The complete, unfiltered results are provided in Supplementary Data 1. Here, we focus our discussion on pathways with the most direct relevance to the neuroendocrine‑immune‑inflammatory axis and cardiac remodeling, which formed the basis of our a priori hypothesis. Genome-wide methylation analysis revealed MBSR induced 7,665 intra-group and 7,791 inter-group DMS, enriched in pathways central to the psychoneuroendocrine-immune axis. This provides a molecular substrate for the anti-inflammatory effect: hypomethylation of innate immune genes (TLR5, CD8A)^27,28^ primes immune surveillance, while hypermethylation of the pro-inflammatory mediator FSTL1^29^ likely suppresses its NF-κB–driven activity. This pattern—enhancing defense while suppressing deleterious inflammation—aligns with the immunomodulatory signature of mind-body interventions^30^ and is reflected in the reduced systemic inflammatory markers.

Beyond inflammation, MBSR induced a protective epigenetic signature against oxidative stress—a known driver of post-AMI damage^10,26^. Hypermethylation of NCF1 (a key subunit of NADPH oxidase)^31^ and hypomethylation of ISCU and HAGH (genes critical for mitochondrial function and antioxidant capacity)^32,33^ suggest a coordinated mechanism to suppress ROS production while enhancing antioxidant capacity. Oxidative stress is closely intertwined with inflammation^34^ and impairs cardiac metabolic efficiency, linking these epigenetic modifications to improved myocardial contractility (GLS) and objective functional capacity (6MWD)^35,36^.

Notably, MBSR’s impact extended to neuroendocrine regulation: differential methylation was identified in HPA axis-related genes (e.g., NR3C1, the glucocorticoid receptor)^37^, with enrichment in neuroactive ligand-receptor interaction pathways. This epigenetic modulation likely normalizes stress-responsive neuroendocrine circuits^38^, restoring negative feedback sensitivity and interrupting the maladaptive “stress-inflammation-oxidative stress” cascade^11^. which links stress reduction to improved cardiac structure/function (evidenced by better LVEF and 6MWD)^39^.

Additionally, MBSR-induced hypermethylation of coagulation factors (F5, F11)^40^, and methylation changes in the Wnt/β-catenin pathway (e.g., WNT4)^41^, suggest a dual epigenetic strategy to attenuate thrombotic risk and pro-fibrotic signaling. This is structurally corroborated by reduced interventricular septal thickness and improved wall motion score index (WMSI) in the MBSR group, confirming attenuated adverse cardiac remodeling.

Integration of our methylome with an external coronary artery disease transcriptome prioritized candidate hub genes (BRD4, PTTG1IP). Exploratory correlation analysis, while not passing strict multiple comparison correction, provided hypothesis-generating clues: BRD4 methylation correlates with lower CRP and better GLS, and PTTG1IP methylation with higher LVEF. Given their known roles in inflammatory signaling and cellular regulation^42,43^, these genes may serve as epigenetic integrators and candidate nodes in the “epigenetic-inflammatory-cardiac functional axis”, a concept supported by the role of relaxation responses in modulating relevant biological networks^44^.

Limitations include the non-randomized study design, modest overall sample size, and particularly small subgroup for DNA methylation analysis (*n* = 5 per group), as well as the all-male cohort—all factors that necessitate cautious interpretation and underscore the exploratory, hypothesis-generating nature of this pilot study. The all-male enrollment resulted from eligible female participants’ refusal to join the MBSR program (12 declined upfront, 3 withdrew after the first session), which precludes sex-specific subgroup analysis and severely limits generalizability to female patients. This is particularly critical given well-documented sex differences in post-AMI inflammatory responses, cardiac remodeling, psychological stress reactivity, and epigenetic regulation. Therefore, our findings should not be extrapolated to women without direct, prospective validation in female cohorts. Future studies should prioritize large-scale randomized controlled trials with adequate female representation, potentially employing tailored recruitment and retention strategies (e.g., female-only MBSR groups, flexible scheduling to accommodate caregiving responsibilities) to formally test for sex-specific effects and determine whether MBSR confers similar cardioprotection in female post-PCI patients. While our functional inferences are strongly supported by pathway enrichment and external transcriptomic validation, the absence of in-house transcriptomic data from the same cohort requires direct, prospective confirmation in future investigations.

## Conclusions

This pilot study provides preliminary evidence that an 8‑week MBSR program induces widespread DNA methylation changes in post‑PCI AMI patients, which are associated with reduced systemic inflammation and improved cardiac and functional outcomes. The enrichment of differentially methylated genes in neuroendocrine, inflammatory, and oxidative stress pathways suggests a potential epigenetic mechanism linking stress reduction to cardioprotection. While exploratory and requiring validation in larger randomized trials, these findings offer a mechanistic framework for integrating MBSR into post‑infarction care.

## Electronic Supplementary Material

Below is the link to the electronic supplementary material.

Supplementary Material 1

Supplementary Material 2

Supplementary Material 3

## Data Availability

The DNA methylation sequencing data generated in this study have been deposited in the NCBI Gene Expression Omnibus (GEO) database under accession number GSE317022. The data will be made publicly available upon publication of this manuscript. The permanent link is: https://www.ncbi.nlm.nih.gov/geo/query/acc.cgi?acc=GSE317022.
