# Supplementary material for: DNA methylation mediates MBSR induced cardioprotection in patients after PCI
Source: Sci Rep. 2026 May 9;16:21293. doi: 10.1038/s41598-026-51616-6 (PMC13346535; doi:10.1038/s41598-026-51616-6)
Supplement: Supplementary file 3 — Supplementary Material 3 [file 41598_2026_51616_MOESM3_ESM.pdf]

Supplementary Information

DNA Methylation Mediates MBSR induced Cardioprotection in Patients after PCI

Mengyuan Xiong[1†], Xingkui Dou[2†], Jifa Tao[1], Fei Hu[1], Pan Jing[1], Zhao Zhao[1], Hongyan Cai[1], Zhao Hu[3*], and Min Zhang[1*]

1Cardiology Department, the First Affiliated Hospital of Kunming Medical University, Kunming 650032, China

2Cardiology Department, the Second Affiliated Hospital of Kunming Medical University, Kunming 650101, China

3Geriatric Cardiology Department, the First Affiliated Hospital of Kunming Medical University, Kunming 650032, China

†Drs. Xiong and Dou contributed equally to this work as co-first author.

*Correspondence: Min Zhang (Zhangm@ydyy.cn), and Zhao Hu (Huzhao@ydyy.cn)

Section 1: Supplementary Methods

1. Detailed Procedures for Blood Sample Processing and Inflammatory Marker Assays

Peripheral venous blood (5 mL) was drawn from each participant into EDTA-coated vacutainers between 9:00 and 10:00 AM after a 12-hour fast and 48-hour abstinence from alcohol. Samples were immediately centrifuged at 3,000 × g for 10 minutes at 4°C to separate plasma. Plasma aliquots were stored at -80°C until analysis.

Inflammatory markers were quantified as follows:

- C-reactive protein (CRP): Measured by immunoturbidimetric assay using the CRP Gen.3 kit on a cobas c 502 analyzer (Roche Diagnostics, Mannheim, Germany).
- Interleukin-6 (IL-6): Quantified using the Human IL-6 Quantikine ELISA Kit (R&D Systems, Minneapolis, MN, USA; Catalog No. D6050) according to the manufacturer's instructions. Absorbance was read at 450 nm with wavelength correction at 540 nm.
- Procalcitonin (PCT): Measured via a chemiluminescent immunoassay on an ADVIA Centaur XP system (Siemens Healthineers, Erlangen, Germany).

2. Detailed Protocols for DNA Methylation Profiling (RRBS)

2.1. DNA Extraction and Quality Control

Genomic DNA was isolated from buffy coat cells using the QIAamp DNA Blood Mini Kit (Qiagen, Hilden, Germany; Catalog No. 51104), following the manufacturer's "Purification of Total DNA from Blood or Body Fluids" protocol. DNA concentration and purity were assessed using a NanoDrop 2000 spectrophotometer (Thermo Fisher Scientific). DNA integrity was verified by 1% agarose gel electrophoresis. Only samples with an A260/A280 ratio between 1.8 and 2.0 and clear, high-molecular-weight bands were used for library construction.

2.2. Reduced Representation Bisulfite Sequencing (RRBS) Library Preparation

RRBS libraries were prepared according to a published protocol [1] with minor modifications. Briefly:

- Digestion: 200 ng of genomic DNA was digested with the methylation-insensitive restriction enzyme MspI (New England Biolabs) at 37°C for 16 hours.
- End Repair and A-Tailing: Digested fragments were end-repaired, 3'-adenylated, and ligated to methylated Illumina TruSeq adapters using the NEBNext Ultra II DNA Library Prep Kit (New England Biolabs).
- Bisulfite Conversion: Adapter-ligated DNA was treated with the EZ-96 DNA Methylation-Gold Kit (Zymo Research, Irvine, CA, USA) following the standard protocol.
- PCR Enrichment and Clean-up: Converted DNA was PCR-amplified for 12-15 cycles. The final libraries were purified using AMPure XP beads (Beckman Coulter) and quantified by Qubit dsDNA HS Assay Kit (Thermo Fisher Scientific). Fragment size distribution was analyzed on an Agilent 2100 Bioanalyzer.

2.3. Sequencing

Final qualified libraries were pooled in equimolar amounts and sequenced on an Illumina NovaSeq 6000 platform (Illumina, San Diego, CA, USA) in a 150-bp paired-end (PE150) format at Kunming Kingmed Medical Laboratory.

3. Bioinformatics Pipelines and Software Parameters

3.1. DNA Methylation Data Processing

- Quality Control & Trimming: Raw sequencing reads were processed with fastp (version 0.22.0) [2] to remove low-quality bases (Q<20) and adapter sequences.
- Alignment: Clean reads were aligned to the human reference genome (hg38/GRCh38) using Bismark (version 0.23.0) [3] with the Bowtie2 aligner. Duplicate reads were removed

using deduplicate_bismark.

- Methylation Calling & Differential Analysis: Methylation levels at individual CpG sites were extracted using the bismark_methylation_extractor function. Differential methylation analysis was performed in R (version 4.2.0) using the DSS package (version 2.46.0) [4]. Differentially methylated sites (DMS) and regions (DMR) were identified with thresholds of FDR < 0.05 and an absolute methylation difference ($|\Delta\beta|$) ≥ 3%.

We did not use a published pipeline such as Nextflow; the analysis was performed with a custom workflow using the tools listed above.

3.2. Functional Enrichment and Integrative Analysis

All functional analyses were performed in R.

- Annotation: DMS/DMR were annotated to genomic features (promoters, gene bodies, etc.) using the ChIPseeker package (version 1.34.0) [5].
- Pathway Enrichment: Gene Ontology (GO) and Kyoto Encyclopedia of Genes and Genomes (KEGG) pathway enrichment analyses were conducted using the clusterProfiler package (version 4.4.4) [6]. Terms with an adjusted P-value (FDR) < 0.05 were considered significantly enriched.
- Protein-Protein Interaction (PPI) Network: Gene lists of interest were submitted to the STRING database (version 11.5) [7] to retrieve interaction networks with a minimum required interaction score of 0.4 (medium confidence). Networks were visualized and analyzed using Cytoscape (version 3.9.1) [8].

4. Statistical Analysis Software

All statistical analyses for clinical data were performed using R (version 4.2.0) or SPSS Statistics (version 26.0, IBM Corp.). Specific R packages used included lme4 for GLM and stats for ANCOVA/t-tests. Figures were generated using ggplot2 (version 3.4.0).

References for Supplemental Methods

[1] Nakabayashi K, Yamamura M, Haseagawa K, Hata K: Reduced Representation Bisulfite Sequencing (RRBS). Methods Mol Biol 2023, 2577:39-51.

[2] Chen S, Zhou Y, Chen Y, Gu J: fastp: an ultra-fast all-in-one FASTQ preprocessor. Bioinformatics 2018, 34(17):i884-i890.

[3] Krueger F, Andrews SR: Bismark: a flexible aligner and methylation caller for Bisulfite-

Seq applications. Bioinformatics 2011, 27(11):1571-1572.
[4] Park Y, Wu H: Differential methylation analysis for BS-seq data under general experimental design. Bioinformatics 2016, 32(10):1446-1453.
[5] Yu G, Wang LG, He QY: ChIPseeker: an R/Bioconductor package for ChIP peak annotation, comparison and visualization. Bioinformatics 2015, 31(14):2382-2383.
[6] Yu G, Wang LG, Han Y, He QY: clusterProfiler: an R package for comparing biological themes among gene clusters. OMICS 2012, 16(5):284-287.
[7] Szklarczyk D, Gable AL, Nastou KC, Lyon D, Kirsch R, Pyysalo S, Doncheva NT, Legeay M, Fang T, Bork P et al: The STRING database in 2021: customizable protein-protein networks, and functional characterization of user-uploaded gene/measurement sets. Nucleic Acids Res 2021, 49(D1):D605-D612.
[8] Shannon P, Markiel A, Ozier O, Baliga NS, Wang JT, Ramage D, Amin N, Schwikowski B, Ideker T: Cytoscape: a software environment for integrated models of biomolecular interaction networks. Genome Res 2003, 13(11):2498-2504.

Section 2: Supplementary Tables

Supplementary Table S1. MBSR Program Curriculum and Homework Assignments

Time	Week 1	Week 2	Week 3	Week 4	Week 5	Week 6	Week 7	Week 8
Curriculum	Awareness	Perception	Present Focus	Stress Response	Acceptance	Thoughts & Emotions	Depleting & Nourishing	Practice
Homework	Mindful Breathing	Body Scan	Mindful Yoga	Mindful Walking	Mindful Eating	Listening & Understanding	Group Sharing	Compassion -Meditation

Note: Each weekly curriculum session was approximately 2–2.5 hours, focusing on specific mindfulness techniques adapted from Kabat-Zinn. Homework assignments were 30-minute daily audio-guided exercises to reinforce in-session learning.

Supplementary Table S2. Thematic Domains and Subthemes of Semi-Structured Interviews

Thematic Domains	Subthemes
1. Quality of Life (QoL)	• Emotional well-being: Reduction in anxiety and depression<br>• Physical health: Alleviation of somatic symptoms and improvement in sleep quality<br>• Social functioning: Enhancement of interpersonal relationships and perceived social support<br>• Healthcare experience: Improved disease understanding, medication adherence, and satisfaction with medical care
2. Mindfulness Skills	• Enhanced present-moment awareness<br>• Improved concentration and attention regulation
3. Self-Regulatory Capacity	• Cognitive regulation: Development of more adaptive thinking patterns<br>• Emotional regulation: Enhancement of coping strategies for negative emotions<br>• Behavioral regulation: Promotion of healthy habit formation

Supplementary Table S3. Characteristics of Subjects at Baseline

Characteristics	MBSR group (n=17)	Control group (n=34)	Statistic	P-value
Male [n(%)]	17（100%）	34（100%）	/	1
Age (y, Mean ± SD)	53.6 ± 6.1	54.9 ± 9.2	-0.507	0.614
BMI (kg/m²)	25.5 ± 3.5	25.3 ± 3.7	0.206	0.838
Married[n(%)]	17（100%）	34（100%）	/	1
Education level [n(%)]			0.661	0.719
High School	9（53%）	19（56%）		
Associate Degree	4（24%）	10（29%）		
Bachelor's Degree	4（24%）	5（15%）		
Current smoking [n(%)]	12 (71%)	25 (74%)	0.049	0.524
Heavy drinker [n(%)]	6（35%）	11（32%）	0.044	0.834
Hypertension [n(%)]	5（29%）	18（53%）	2.534	0.111
Diabetes [n(%)]	5（29%）	8（24%）	/	0.738
MI type [n(%)]			/	0.503
STEMI	12（71%）	27（79%）		
NSTEMI	5（29%）	7（21%）		
Number of diseased vessels [n(%)]			0.049	0.976
Single-vessel	6（35%）	11（32%）		
Two-vessel	3（18%）	6（18%）		

Three-vessel	8（47%）	17（50%）		
Culprit vessel [n (%)]			1.593	0.451
LAD	10（59%）	16（47%）		
LCX	3（18%）	4（12%）		
RCA	4（24%）	14（41%）		
Medication [n(%)]				
Aspirin	17（100%）	34（100%）	/	1
P2Y12 receptor antagonists	17（100%）	34（100%）	/	1
Beta blockers	14（82%）	24（71%）	0.826	0.363
ARNI/ACEI/ARB	13（76%）	21（62%）	1.103	0.294
Statins	17（100%）	34（100%）	/	1
CCBs	2（12%）	7（21%）	/	0.699
Anticoagulants	0（0%）	0（0%）		/
GP IIb/IIIa inhibitor	3（18%）	20（59%）	7.761	0.005**
IABP [n(%)]	2（12%）	2（6%）	/	0.593
Left ventricular aneurysm [n(%)]	3（18%）	4（12%）	/	0.673
Thromboembolism [n(%)]	0（0%）	0（0%）	/	/
Killip's grade at admission [n(%)]			0.916	0.632
Grade I	11（65%）	26（76%）		
Grade II	4（24%）	6（18%）		
Grade III	0（0%）	0（0%）		
Grade IV	2（12%）	2（6%）		

Notes：

1. Continuous variables with normal distribution: t-test; non-normal: Mann-Whitney U test. Categorical variables: chi-squared (χ^2) or Fisher's exact test. *P<0.05, **P<0.01 for significance.

BMI: body mass index; STEMI: ST-segment elevated myocardial infarction; NSTEMI: non-ST-segment elevated myocardial infarction; LAD: left anterior descending artery; LCX: left circumflex artery; RCA: right coronary artery; ARNI: angiotensin receptor neprilysin inhibitor; ACEI: angiotensin-converting enzyme inhibitor; ARB: angiotensin receptor blockers; CCBs: calcium channel blockers; GP: glycoprotein; IABP: intra-aortic balloon pump

Supplementary Table S4. Summary of Differentially Methylated Sites and Regions Identified by RRBS under Nominal and Genome-wide Corrected Thresholds

Comparison	Threshold	DMS (n)	Genomic Distribution (Top 3)	DMR (n)	Genomic Distribution (Top 3)
Intra-group (post-MBSR vs pre-MBSR)	Nominal: FDR < 0.05, |Δ| ≥ 3%	7,665	Intron (49%), Intergenic (40%), Exon (5%)	206	Intron (43%), Intergenic (43%), Exon (8%)
	Genome-wide FDR<0.05	0	-	0	-
Inter-group (post-MBSR vs control)	Nominal: FDR < 0.05, |Δ| ≥ 3%	7,791	Intron (50%), Intergenic (40%), Exon (5%)	217	Intron (51%), Intergenic (35%), Exon (7%)
	Genome-wide FDR<0.05	0	-	0	-

Supplementary Table S5. KEGG pathways with nominal enrichment (raw P < 0.1) in intra-group and inter-group comparisons

Comparison	Pathway ID	Pathway Name	Gene_Count	Background_Count	raw P-value	padj
Intra-group (post-MBSR vs pre-MBSR)	hsa05340	Primary immunodeficiency	2	38	0.015	0.6607
	hsa04660	T cell receptor signaling pathway	2	103	0.092	0.6607
	hsa04080	Neuroactive ligand-receptor interaction	4	353	0.0958	0.6607
Inter-group (post-MBSR vs control)	hsa04918	Thyroid hormone synthesis	3	75	0.0136	0.4242
	hsa05224	Breast cancer	4	147	0.0165	0.4242
	hsa05226	Gastric cancer	4	148	0.0169	0.4242
	hsa04610	Complement and coagulation cascades	3	85	0.019	0.4242
	hsa04310	Wnt signaling pathway	4	167	0.0251	0.4242

Notes: Pathways were prioritized based on raw P < 0.1 and biological relevance to the epigenetic-inflammatory-cardiac functional axis. After Benjamini-Hochberg false discovery rate (FDR) correction for multiple comparisons, no KEGG pathway reached statistical significance (all padj > 0.05). The complete, unfiltered KEGG enrichment results for all comparisons are available in Supplementary Data 1 (Excel file) .

Supplementary Table S6. GO terms with nominal enrichment (raw P < 0.1) in intra-group and inter-group comparisons

Comparison	GO_ID	Term	Category	Gene_Count	Background_Count	raw P-value	padj
Intra-group (post-MBSR vs pre-MBSR)	GO:0030488	tRNA methylation	BP	3	42	0.000894	0.5768
	GO:0060263	regulation of respiratory burst	BP	2	17	0.002612	0.5768
	GO:0007269	neurotransmitter secretion	BP	4	149	0.004629	0.5768
	GO:0001510	RNA methylation	BP	3	85	0.006695	0.5768
	GO:0072332	intrinsic apoptotic signaling pathway by p53 class mediator	BP	3	88	0.007366	0.5768
	GO:0006836	neurotransmitter transport	BP	4	222	0.018059	0.5768
	GO:0042554	superoxide anion generation	BP	2	48	0.019794	0.5768
	GO:0001505	regulation of neurotransmitter levels	BP	4	237	0.022342	0.5768
Inter-group (post-MBSR vs control)	GO:0007284	negative regulation of adenylate cyclase activity	BP	2	31	0.02845	0.638
	GO:0006886	intracellular protein transport	BP	3	138	0.03121	0.638
	GO:0032874	positive regulation of stress-activated MAPK	BP	2	59	0.04157	0.638

		cascade					
	GO:0007189	adenylate cyclase-activating G protein-coupled receptor signaling pathway	BP	3	155	0.04372	0.638
	GO:0043491	protein kinase B signaling	BP	3	162	0.04789	0.638
	GO:0006979	response to oxidative stress	BP	5	493	0.07222	0.638
	GO:0031396	regulation of protein ubiquitination	BP	3	174	0.08326	0.638
	GO:0051897	positive regulation of protein kinase B signaling	BP	2	89	0.08935	0.638

Notes: Terms were selected based on raw $P < 0.1$ and biological relevance to the epigenetic-inflammatory-cardiac functional axis. After Benjamini-Hochberg FDR correction for multiple comparisons, no GO term reached statistical significance (all padj > 0.05). The complete, unfiltered GO enrichment results for all comparisons are available in Supplementary Data 1 (Excel file).

Supplementary Table S7. Summary of key genes and modules identified from network-based and multi-omics analyses.

Category	Identified Genes / Modules	Primary Associated Function/Biological Theme
**A. PPI Network Modules**	Immune-Inflammation Module (Core nodes: *TLR5*, *CD8A*, *CAMP*)	Innate & adaptive immune response, T-cell activation, antimicrobial defense.
	Coagulation-Metabolism Module (Core nodes: *F5*, *F11*, *ISCU*, *HPD*)	Coagulation cascade, mitochondrial metabolism, oxidative stress resistance.
	TLR5	Highlighted as a pivotal hub bridging immune and inflammatory pathways.
	F5 and *ISCU*	Exhibited functional cross-talk linking coagulation and mitochondrial apoptosis.
**B. Multi-omics Integration**	Total Prioritized Candidate Hub Genes	25 genes identified via methylome-transcriptome integration.
	Representative Hub Genes (e.g., *WNT4*, *FSTL1*, *NCF1*, *BRD4*, *PTTG1IP*)	Immune regulation, inflammation suppression, oxidative stress, epigenetic regulation.
	Key Sub-network (Inter-group) (e.g., *WNT4*, *FSTL1*, *NCF1*)	Enriched in "oxidative stress response" and "extracellular matrix organization".
	Key Sub-network (Intra-group) (e.g., *BRD4*, *ISCU*, *PTTG1IP*)	Linked to "inflammatory signaling" and "mitochondrial function".
	Venn Analysis (Overlap Counts)	1,829 & 2,063 overlapping genes from hypomethylated/upregulated sets.

Section 3: Supplementary Figures

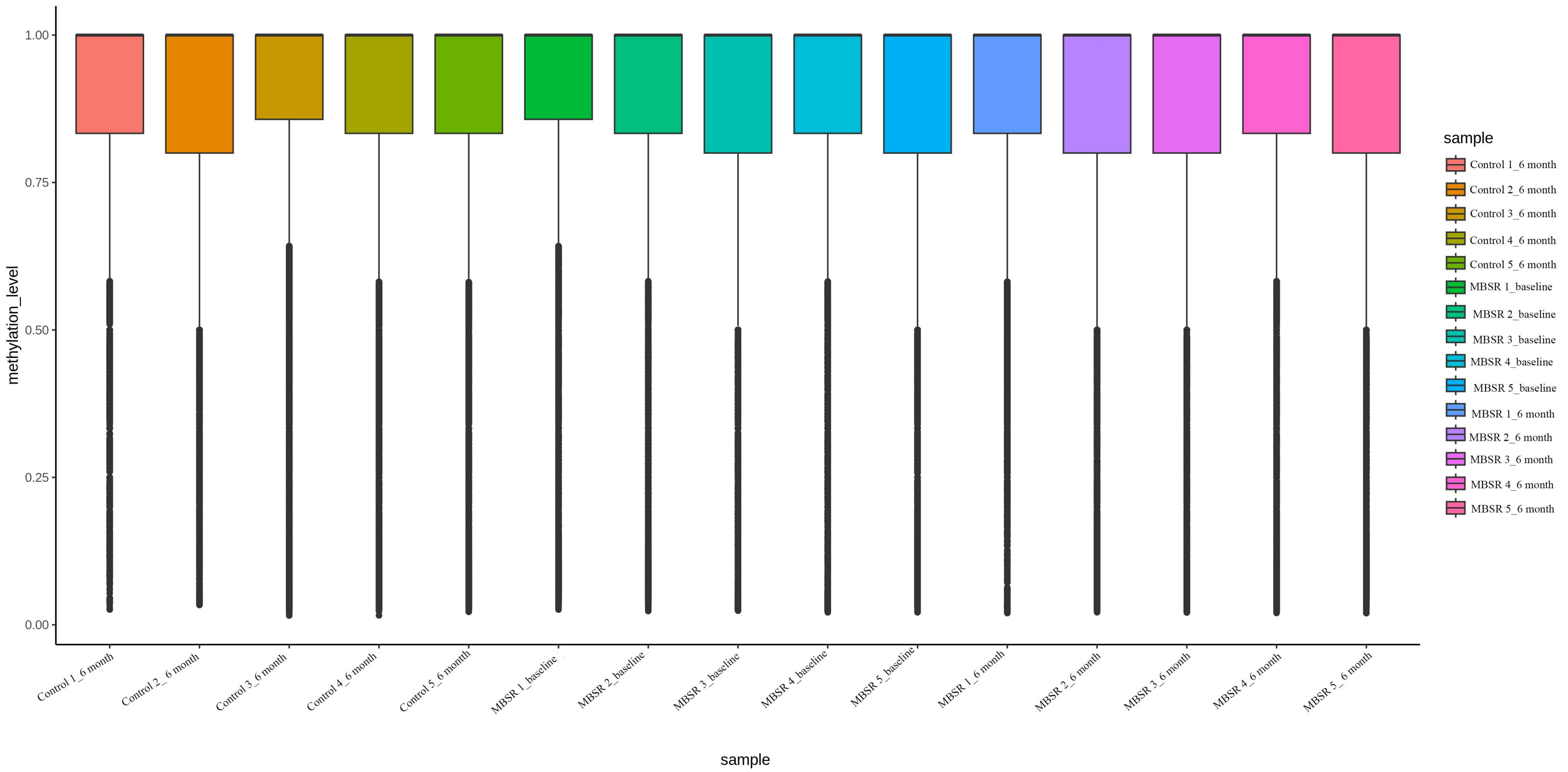

Supplementary Fig. S1. Distribution of DNA methylation Beta values for RRBS-profiled CpG sites

Beta values (0 = fully unmethylated, 1 = fully methylated) are shown for high-quality CpG sites (sequencing depth ≥10×) across all samples, including MBSR baseline (n=5), MBSR 6-month follow-up (n=5), and control 6-month follow-up (n=5). All CpG sites underwent strict quality control, including filtering of low-quality/duplicate reads, adapter sequences, and known SNP-overlapping loci. The characteristic bimodal distribution observed in all subgroups confirms the biological validity of the methylation data, while high intra-group consistency verifies the reliability of the sequencing. The subtle shift in the MBSR 6-month distribution reflects the overall methylome alteration induced by MBSR intervention.

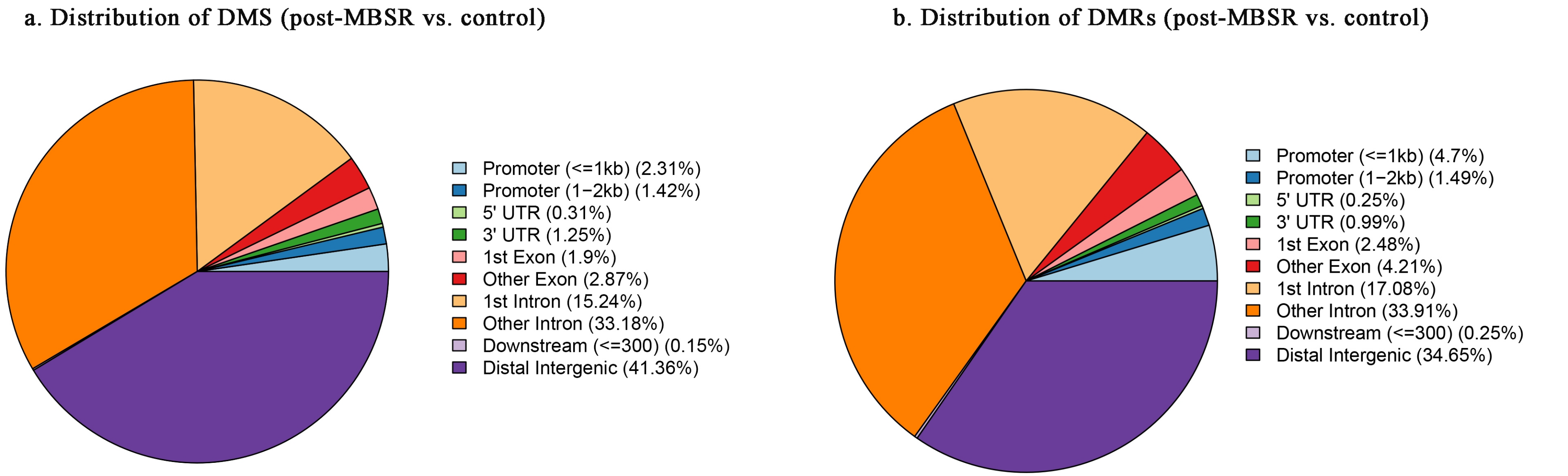

Supplementary Fig. S2. Genomic Distribution of DMS and DMRs Between 6 Months post-MBSR and Control Groups

a. Intra-group (post-MBSR vs. pre-MBSR) GO Enrichment Bar Plot

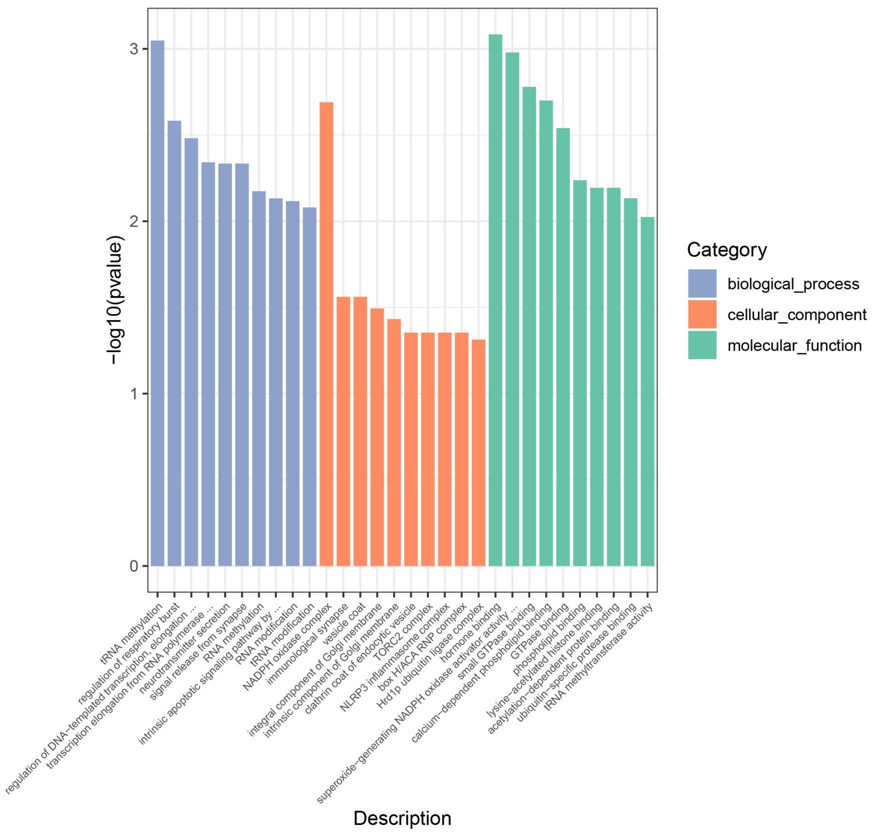

b. Inter-group (post-MBSR vs. control) GO Enrichment Bar Plot

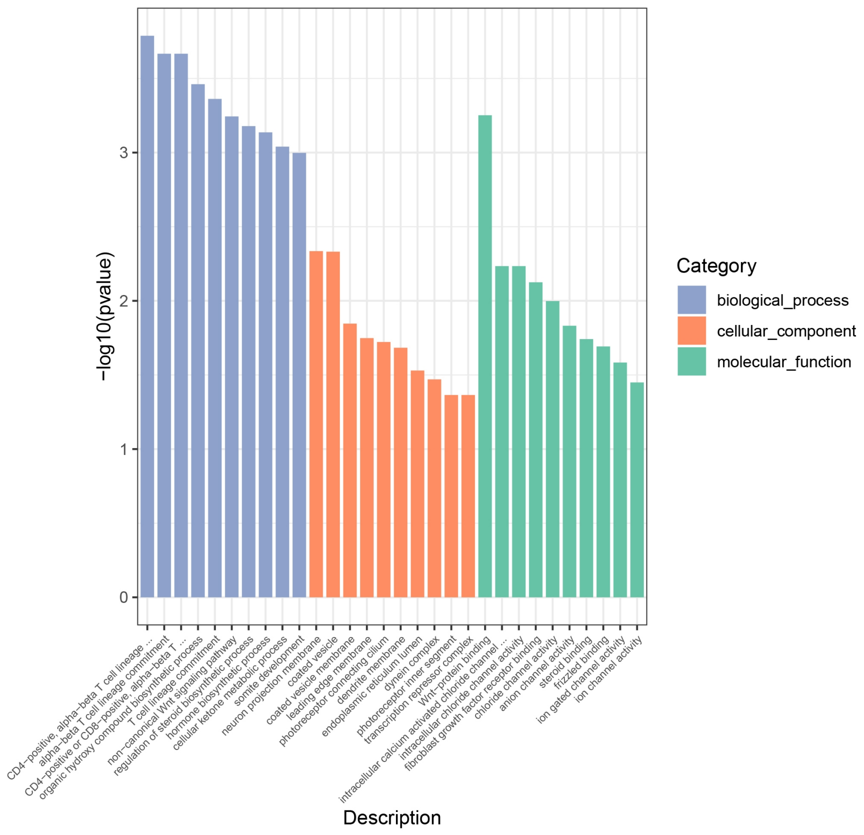

Supplementary Fig. S3. Gene Ontology (GO) Enrichment Analysis

a. Inter-group PPI Network (post-MBSR vs. Control) Enriched in Oxidative Stress and Extracellular Matrix Pathways

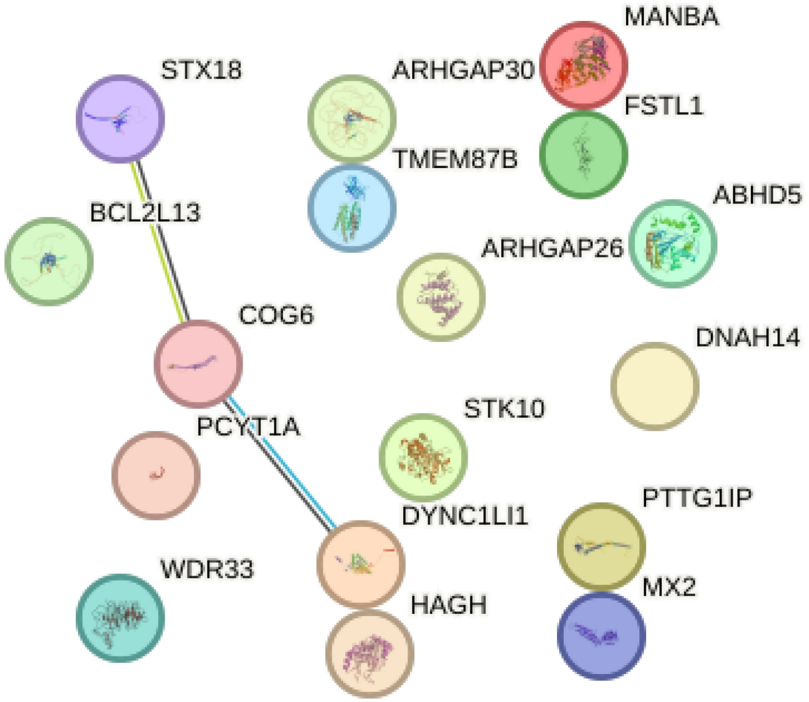

b. Intra-group PPI Network (pre- vs. post-MBSR) Linked to Inflammatory Signaling and Mitochondrial Function.

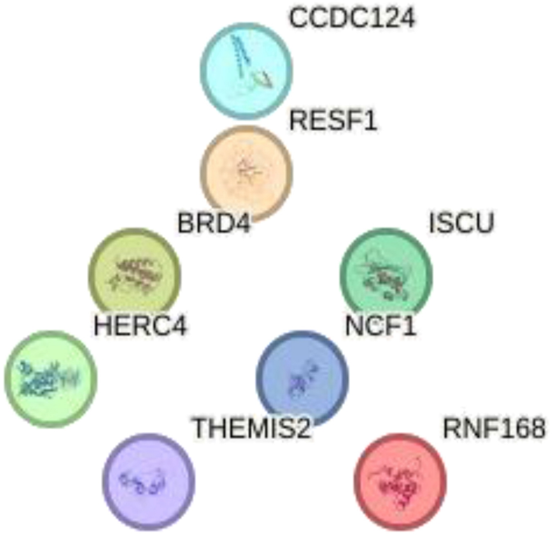

Supplementary Fig. S4. PPI Networks of Epigenomic-Transcriptomic Overlapping Genes

Section 4: Supplementary Data

Supplementary Data 1. Complete KEGG pathway and Gene Ontology (GO) enrichment results (Biological Process, Cellular Component, and Molecular Function) for differentially methylated sites/regions (DMS/DMR). The file includes intra-group (post-MBSR vs. pre-MBSR) and inter-group (post-MBSR vs. control) comparisons, with pathway/term names, gene counts, background counts, raw P-values, and FDR-adjusted P-values (where applicable). All unfiltered results are provided.

Supplementary Data 2. Spearman correlation analysis between DNA methylation levels of 25 hub genes and 11 clinical phenotypes. The table includes correlation coefficients (ρ), raw P-values, and FDR-adjusted P-values (Benjamini-Hochberg method across 275 tests).

Note: Supplementary Data 1 and Supplementary Data 2 are large Excel files and are provided as separate electronic supplementary material. They are not embedded in this PDF due to file size constraints. These files are available for download from the journal's website alongside the article.
